# RNA with Azvudine Incorporated One Nucleotide Upstream of 3’-End Resists Cleavage by SARS-CoV-2 Proofreading Exonuclease

**DOI:** 10.21203/rs.3.rs-9465547/v1

**Published:** 2026-05-25

**Authors:** Ying Tong Yue, Kao Lin, Chuanjuan Tao, Xiaoxu Li, Shuxia Peng, Marianna Teplova, Shiv Kumar, Irina Morozova, Sergey Kalachikov, James J. Russo, Dinshaw J. Patel, Jingyue Ju

**Affiliations:** Columbia University; Columbia University; Columbia University; Columbia University; Memorial Sloan-Kettering Cancer Center; Memorial Sloan-Kettering Cancer Center; Columbia University; Columbia University; Columbia University; Columbia University; Memorial Sloan-Kettering Cancer Center; Columbia University

## Abstract

The cytidine analog Azvudine (Azv) showed promising therapeutic effects against Severe Acute Respiratory Syndrome Coronavirus 2 (SARS-CoV-2), leading to its conditional authorization as a COVID-19 treatment in China. Once Azvudine enters infected cells, it is converted to the active form, Azvudine-triphosphate, which has been shown to inhibit polymerases of several viruses. Here we show that when Azvudine is incorporated into RNA by the SARS-CoV-2 RNA-dependent RNA polymerase (RdRp), it hinders incorporation of the subsequent nucleotide. However, the 3’-Azv-terminated RNA does not show any resistance to the SARS-CoV-2 proofreading exonuclease (ExoN). Furthermore, we found that the incorporation of Azvudine at the penultimate position of the RNA prevents RNA cleavage by the ExoN. These findings may provide molecular insight for the development of novel nucleotide analogs that can inhibit RdRp and resist the viral proofreading mechanism of coronaviruses.

## Introduction

RNA-dependent RNA polymerase (RdRp), the enzyme responsible for RNA synthesis in positive-sense RNA viruses, is one of the most conserved nonstructural proteins in these viruses [1–5]. It is a low fidelity enzyme with no counterparts in human cells, making it an ideal target for antiviral therapeutics. Nucleoside/nucleotide analogs are among the most important RdRp inhibitors. Their active triphosphate forms compete with natural nucleoside triphosphates for RdRp incorporation during viral RNA synthesis. Once incorporated, nucleotide analogs can lead to chain termination or reduction of viral RNA synthesis, as well as lethal mutations [6–8], thus hindering viral replication in the host. Because these nucleoside/nucleotide analogs interact with the enzyme’s highly conserved active site, they have broad-spectrum antiviral potential [9–13].

The SARS-CoV-2 RdRp complex, the fastest viral polymerase complex characterized to date [14], consists of the main functional nonstructural protein 12 (Nsp12) along with Nsp7 and two Nsp8 proteins acting as cofactors [10]. Due to the large viral genome (~ 30kb) and the low fidelity RdRp, SARS-CoV-2 also possesses a proofreading exonuclease complex (ExoN, consisting of Nsp14 and Nsp10) to remove mis-incorporated nucleotides as well as nucleotide analogs in the replicating RNA to maintain its genome integrity [15]. This poses a major challenge to nucleotide analog-based drug development for coronaviruses. For example, studies have revealed that Remdesivir, the first FDA-approved nucleotide analog drug against COVID-19, has no ExoN resistance, partially undermining its antiviral activity and clinical efficacy [16–18].

**Figure 1:**
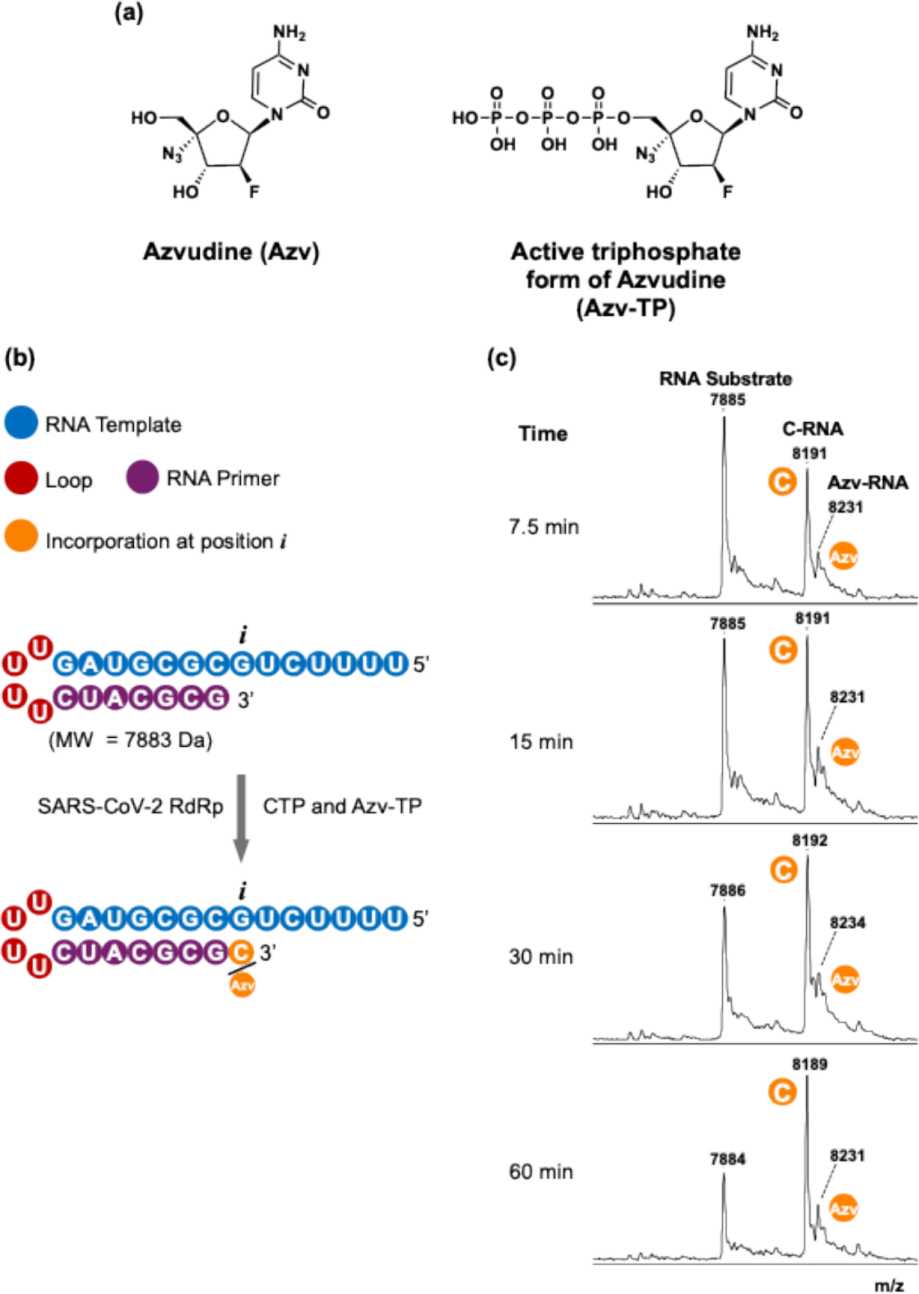
Incorporation of Azv-TP by SARS-CoV-2 RdRp in the presence of CTP. (**a**) Structures of Azvudine and its active triphosphate form (Azv-TP). (**b**) Annealed RNA template-loop-primer substrate (top) and RNA products after RdRp reaction (bottom). (**c**) MS analysis of time-dependent CTP and Azv-TP incorporation by RdRp. Incubation time is indicated to the left of each figure. Azv-TP was incorporated by RdRp in the presence of CTP with 2- to 3-fold lower incorporation when the concentration ratio between CTP and Azv-TP is 1:40.

Azvudine is a cytidine analog carrying a 2′-deoxy-2′-β-fluoro-4′-azido modified ribose that targets viral polymerases and has demonstrated broad-spectrum antiviral activity against human immunodeficiency virus, hepatitis C virus and hepatitis B virus [19–23] (Fig. 1a). Azvudine was demonstrated to be beneficial for hospitalized patients with COVID-19 [24–26], leading to its conditional approval in China. An early study revealed that Azvudine homes to the thymus and functions as an effective COVID-19 antiviral drug [27]. Azvudine has also been shown to have an anti-inflammatory activity in SARS-CoV-2 infected rhesus macaques [28]. While Azvudine was thought to target SARS-CoV-2 RdRp acting as a terminator, recent polymerase kinetics studies have shown that Azv-TP is a poor substrate of SARS-CoV-2 RdRp and cannot compete with its natural nucleotide CTP for RdRp incorporation [29]. Here we show that when Azvudine is incorporated into RNA by SARS-CoV-2 RdRp, it hinders incorporation of the subsequent nucleotide. However, the 3’-Azv-terminated RNA does not show any resistance to the SARS-CoV-2 proofreading exonuclease. Furthermore, we found that when Azvudine is incorporated at the penultimate position of the RNA strand, it prevents RNA cleavage by the SARS-CoV-2 ExoN. These findings may provide molecular insight for the development of novel nucleotide analogs that can inhibit RdRp and resist the viral proofreading mechanism of coronaviruses affording broad-spectrum therapeutic potential, due to the conserved active sites of these enzymes in coronaviruses [30–32].

## Results and Discussion

### The cytidine analog Azvudine-TP (Azv-TP) is incorporated into RNA by SARS-CoV-2 RdRp less efficiently than CTP.

Azv-TP has a cytosine base with a modified ribose ring (Fig. 1a). We first assessed whether Azv-TP can be incorporated by SARS-CoV-2 RdRp using a MALDI-TOF MS-based enzymatic assay using CTP as the control (Fig. S-1a) [33]. In the separate tube experiment, Azv-TP incorporation by SARS-CoV-2 RdRp was significantly less than its natural counterpart CTP at the same concentration as measured by MALDI-TOF MS (Fig. S-1b, c).

To further investigate the relative incorporation ability of Azv-TP in the presence of CTP in RNA elongation by SARS-CoV-2 RdRp, a time course same tube experiment was performed, in which CTP and Azv-TP were mixed at a 1:40 ratio. A 25-nucleotide template-loop-primer RNA was used as a substrate for CTP or Azv-TP incorporation (Fig. 1b). Taking advantages of the high resolution MALDI-TOF MS assay, we were able to differentiate the C-/Azv-incorporated RNA products based on their different molecular weight. As shown in Fig. 1c, incorporation of Azv-TP in the presence of CTP was evidenced by the appearance of peak at 8233 Da, which corresponds to the Azv-incorporated RNA product (Azv-RNA) next to the 8189 Da peak, which corresponds to the C- incorporated RNA product (C-RNA). Both C-RNA (~ 8191 Da) and Azv-RNA (~ 8231 Da) peaks showed a time-dependent increase with comparable decreases of the initial RNA substrate (~ 7885 Da) (Fig. 1c). The two peaks maintained a 2 ~ 3-fold difference at all time points, indicating that Azv-TP was incorporated ~ 100 times less efficiently than CTP in this same tube experiment. This lower incorporation could be ascribed to the lack of a hydroxyl group at the 2’-position and the presence of the 4’-azido group on the ribose of Azv-TP (Fig. 1a).

### Azv significantly inhibits incorporation of the subsequent nucleotide.

**Figure 2:**
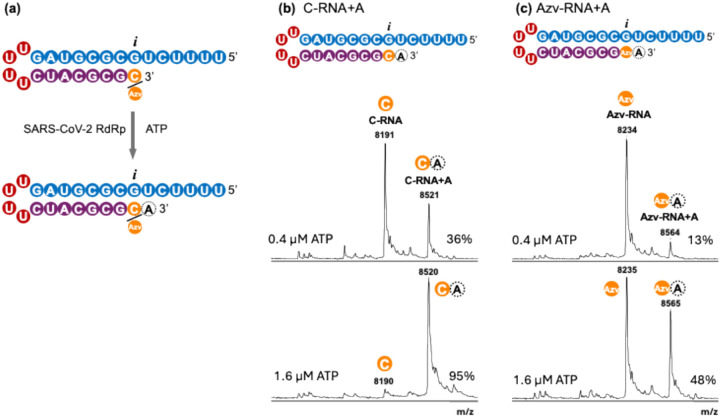
RNA extended by Azv-TP significantly inhibits subsequent nucleotide incorporation by SARS-CoV-2 RdRp. (**a**) Annealed Azv- or C-extended RNA substrates (top) and the single nucleotide extended RNA products (bottom) after RdRp reaction. MS analysis of subsequent ATP incorporation after natural C (**b**) or Azv (**c**) by RdRp. Azv incorporation leads to RNA elongation inhibition when compared to natural RNA elongation, shown by lower Azv-RNA+A (~8564 Da) peak compared to C-RNA+A (~8520 Da) peak. Percentage of ATP incorporation is shown at the lower right of each MS figure.

Azv has been reported to inhibit RNA/DNA synthesis in numerous viruses [19–23] and Lee et al. recently demonstrated that Azv caused chain termination for SARS-CoV-2 RdRp reaction [29]. To investigate the termination effect of Azv in RNA synthesis by SARS-CoV-2 RdRp, Azv-RNA and C-RNA with Azv and cytidine at the 3’-end (position *i*) were used as substrates in the polymerase reaction (Fig. 2a). The effect of incorporated Azv on further RNA extension was determined by the amount of RNA products extended with ATP at the subsequent (*i + 1*) position. When 0.4 μM ATP was added to each RdRp reaction, ~ 36% of C-RNA was extended, while only ~ 13% of Azv-RNA was extended (Fig. 2b, c). At 1.6 μM ATP, nearly all C-RNA (> 95%) was extended with ATP, while only ~ 48% of Azv-RNA was extended. We also showed that Azv hindered subsequent base incorporation in a time-dependent manner (Fig. S-2). The lower incorporation of the nucleotide following Azv suggests that Azv functions as a non-obligate terminator for SARS-CoV-2 RdRp catalyzed RNA elongation and can be partially overcome by high concentrations of the subsequent nucleotide, which is consistent with the result from Lee et al. [29].

To investigate whether Azv could hinder RNA synthesis beyond the *i + 1* position, an excess of 5 μM of ATP and GTP (based on the complementary sequence of the template RNA) were mixed with C-RNA or Azv-RNA to overcome the termination effect at the *i* position (Fig. S-3a). As shown in Fig. S-3b, the natural C-RNA showed full extension with no evidence of RdRp stalling (C-RNA + 6). In contrast, Azv-RNA extension resulted in a major product of 5 nucleotides being incorporated (Azv at position *i*, Fig. S-3c), with a significant portion of Azv-RNA (~ 20%) remaining unreacted. These results showed that Azv acts as a non-obligate terminator in RNA elongation that slows down the overall RNA synthesis.

### Azv is excised by ExoN when positioned at the 3’-end of the RNA but resists excision when located one nucleotide upstream of the 3’-end.

**Figure 3:**
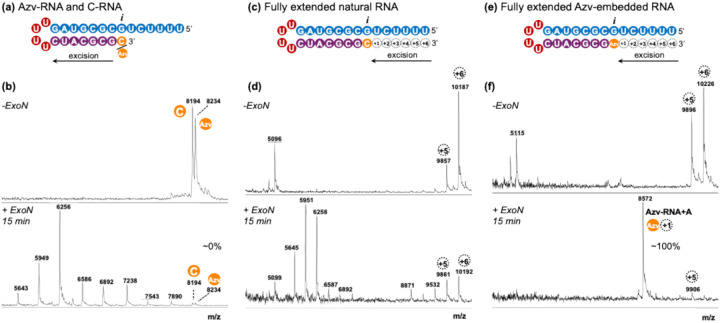
SARS-CoV-2 ExoN excision of Azv-RNA and fully extended Azv-embedded RNA. (**a**) Azv- or C-RNA substrate. ExoN excision direction is indicated by the arrow. (**b**) MS analysis of the RNA mixture before (-ExoN) and after ExoN reaction. Azv-RNA (~8234 Da) showed no ExoN resistance similar to the natural C-RNA. (**c**) Fully extended natural RNA substrate. (**d**) MS analysis of natural RNA ExoN excision as a control showed no ExoN resistance. (**e**) Fully extended Azv-embedded RNA substrate. (**f**) ExoN excision of fully extended Azv-embedded RNA stops at Azv+1 position generating Azv-RNA+A (~8572 Da) which almost completely resists ExoN. Percentage of ExoN resistance of Azv-RNA and Azv-RNA+A are shown by the corresponding peaks in the MS figure.

To determine whether RNA terminated with Azv can resist proofreading ExoN cleavage, we characterized the RNA products of the ExoN reaction using the MALDI-TOF MS-based assay. A mixture of C-RNA (~ 8194 Da) and Azv-RNA (~ 8234 Da) was incubated with ExoN, yielding the cleavage profile shown in Fig. 3a, b. The similar decrease in both RNA substrate peaks indicates that Azv, when located at the 3’-end of the RNA, does not resist ExoN excision.

**Figure 4:**
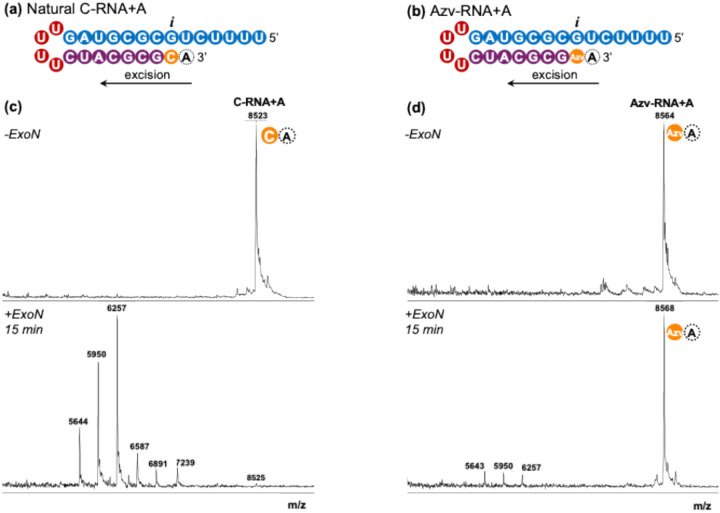
Strong SARS-CoV-2 ExoN resistance of Azv-RNA+1 with A at the *i+1* position. **(a**) Natural C-RNA+A used as a control. (**b**) Azv-RNA+A substrate. ExoN excision direction is indicated by the arrow. (**c**) MS analysis of natural C-RNA+A (~8523 Da) before (-ExoN) and after ExoN reaction indicating no ExoN resistance. (**d**) MS analysis of Azv-RNA+A before (-ExoN) and after ExoN reaction showing that Azv-RNA+A (~8568 Da) has strong ExoN resistance.

However, the polymerase reaction results in Figs. 1 and 2 combined with the ExoN reaction results in Fig. 3b are insufficient to explain Azvudine’s antiviral activity for SARS-CoV-2 infection [24–26]. We therefore performed the following experiments to explore whether the elongated RNA with embedded Azv displays any ExoN resistance. Fully extended natural RNA (Fig. 3c) and Azv-embedded RNA (Fig. 3e) were produced using the same RNA substrate described in Fig. S-3a for the ExoN excision experiments and the products were analyzed by MALDI-TOF MS. As shown in Fig. 3d, all natural RNAs were cleaved to the end of the double-stranded portion of the RNA substrate, whereas cleavage of the Azv-embedded RNA halted at the *i + 1* position where Azv is at position *i*, generating an RNA product with the sequence of 5’-RNA-Azv-A-3’ (Azv-RNA + A) (~ 8572 Da, Fig. 3f). To further examine the ExoN resistance of this specific RNA, Azv-RNA + A and C-RNA + A were produced and incubated with ExoN (Fig. 4a, b). While the natural C-RNA + A was completely cleaved by ExoN (Fig. 4c), Azv-RNA + A showed almost complete ExoN resistance as indicated by the negligible degraded RNA peaks (Fig. 4d). Finally, to investigate whether the resistance of Azv is affected by the specific nucleotide at the *i + 1* position, RNAs with the other three nucleotides (C, G, U) at the *i + 1* position were produced and incubated with ExoN (Fig. S-4). The results indicated that RNAs with Azv incorporated at position *i*, followed by C, G, or U at the *i + 1* position, also showed high resistance to ExoN cleavage (Fig. S-4). These results demonstrated that RNA with Azvudine incorporated one nucleotide upstream of 3’-end strongly resists cleavage by SARS-CoV-2 proofreading exonuclease.

Our biochemical study analyzed the inhibitory effect of Azvudine against SARS-CoV-2 RdRp and the resistance of Azv-contained RNA against the proofreading ExoN. We showed that Azv-TP can be incorporated by SARS-CoV-2 RdRp into the viral RNA at high concentrations in the presence of its natural counterpart CTP. Our result support that the Azv nucleotide acts as a non-obligate terminator that hinders further RNA extension [29]. Notably, we found that the ExoN resistance of Azv-embedded RNA is strongly dependent on the position of the incorporated Azv. When Azv is located at the 3’-end, the RNA has no ExoN resistance, whereas when Azv is located at the penultimate position from the 3’-end, the RNA exhibits near-complete resistance to ExoN cleavage. These findings may provide molecular insight to the development of novel nucleotide analogs that can simultaneously inhibit RdRp and resist the viral proofreading mechanism of coronaviruses due to the conserved coronavirus RdRp and ExoN active sites [30–32, 34], affording broad-spectrum therapeutic potential.

## Methods

RNA oligonucleotides (Template-Loop-Primers) were purchased from Dharmacon. Azvudine triphosphate was purchased from Arctom Scientific. NTPs were purchased from Thermo Fisher Scientific. Other chemicals were purchased from Fisher Scientific or Sigma-Aldrich. The RdRp and ExoN of SARS-CoV-2 were cloned and purified as described [33].

### Primer extension reactions with Azvudine-TP or CTP in separate tubes.

The RNA template-loop-primer (sequences shown in Fig. S-1a) was annealed by heating to 75°C for 3 min and cooling to room temperature in 1x RdRp buffer. Then, 5 μL of 4 μM SARS-CoV-2 Nsp12/Nsp7/Nsp8 (RdRp complex) was added to 5 μL annealed RNA template-loop-primer solution (4 μM) and incubated at room temperature for 5 min. Finally, 10 μL of different concentrations of CTP or Azvudine-TP (0.8 and 1.6 μM) in 1x RdRp buffer were added to the mixture of RdRp and RNA templateloop-primer and incubated at 30°C for 2 hr. The 20 μL extension reaction buffer contained 1 μM RNA template-loop-primer, 1 μM SARS-CoV-2 RdRp complex, and different concentrations of CTP or Azvudine-TP (0.4 and 0.8 μM). The 1x RdRp reaction buffer contained 10 mM pH = 8 Tris-HCl, 10 mM KCl, 2 mM MgCl_2_ and 1 mM β-mercaptoethanol. Desalting of the reaction was performed with an Oligo Clean & Concentrator kit (Zymo Research), resulting in ~ 10 μL purified aqueous RNA solutions. 1 μL of each solution was subjected to MALDI-TOF MS (Bruker ultrafleXtream) analysis. The remaining ~ 9 μL RNA products were used to test exonuclease activity and to perform further elongation.

### Primer extension reactions with Azvudine-TP and CTP in the same tube.

The RNA template-loop-primer (sequences shown in Fig. 1b) was annealed by heating to 75°C for 3 min and cooling to room temperature in 1x RdRp buffer. Then, 5 μL of 4 μM SARS-CoV-2 Nsp12/Nsp7/Nsp8 (RdRp complex) was added to 5 μL annealed RNA template-loop-primer solution (4 μM) and incubated at room temperature for 5 min. Finally, 10 μL of 2 μM CTP and 80 μM Azvudine-TP in 1x RdRp buffer were added to the mixture of RdRp and RNA template-loop-primer and stopped at 7.5, 15, 30, and 60 min. The 20 μL extension reaction buffer contained 1 μM RNA template-loop-primer, 1 μM SARS-CoV-2 RdRp complex, and 1 μM CTP and 40 μM Azvudine-TP. The reactions were quenched with 2.2 μL 100 mM EDTA at 7.5, 15, 30, and 60 min. Desalting of the reaction was performed with an Oligo Clean & Concentrator kit (Zymo Research), resulting in ~ 10 μL purified aqueous RNA solutions. 1 μL of each solution was subjected to MALDI-TOF MS (Bruker ultrafleXtream) analysis.

### Production of Azvudine-terminated RNAs.

Different RNA template-loop-primers (5’-UUUUCUGCGCGUAGUUUUCAUCGCG-3’, 5’- UUUUCAGCGCGUAGUUUUCAUCGCG-3’, 5’-UUUUCCGCGCGUAGUUUUCAUCGCG-3’ or 5’- UUUUCGGCGCGUAGUUUUCAUCGCG-3’) were annealed by heating to 75°C for 3 min and cooling to room temperature in 1x RdRp buffer. Then, 5 μL of 4 μM SARS-CoV-2 Nsp12/Nsp7/Nsp8 (RdRp complex) was added to 5 μL annealed RNA template-loop-primer solution (10 μM) and incubated at room temperature for 5 min. Finally, 10 μL 256 μM Azvudine-TP in 1x RdRp buffer were added to the mixture of RdRp and RNA template-loop-primer and incubated at 30°C for 2 hr. The 20 μL extension reaction buffer contained 1 μM RNA template-loop-primer, 1 μM SARS-CoV-2 RdRp complex, and 128 μM Azvudine-TP. Desalting of the reaction was performed with an Oligo Clean & Concentrator kit (Zymo Research), resulting in ~ 10 μL purified aqueous RNA solutions. 1 μL of each solution was subjected to MALDI-TOF MS (Bruker ultrafleXtream) analysis. The remaining ~ 9 μL RNA products were used to test exonuclease activity or to perform further elongation.

### Single base elongation of C- or Azv-extended RNA.

The C-terminated RNA or Azvudine-terminated RNA (sequences shown in Fig. 2a) was annealed by heating to 75°C for 3 min and cooling to room temperature in 1x RdRp buffer. Then, 5 μL of 4 μM SARS-CoV-2 Nsp12/Nsp7/Nsp8 (RdRp complex) was added to 5 μL of C-terminated RNA (C-RNA) or Azv-terminated RNA (Azv-RNA) (4 μM) and incubated at room temperature for 5 min. Finally, 10 μL of ATP (0.8 μM and 3.2μM for incorporation inhibition test shown in Fig. 2; 20 μM for time dependent subsequent nucleotide hindrance test shown in Fig. S-2) for the next base incorporation in 1x RdRp buffer was added into the mixture of RdRp and terminated RNA and incubated at 30°C for 2 h (Fig. 2) or stopped at 7.5, 15, 30, and 60 min for time dependent reactions (Fig. S-2). The 20 μL extension reaction buffer contained 1 mM terminated RNA, 1 μM SARS-CoV-2 RdRp complex, and different concentrations of ATP (0.4 μM and 1.6 μM for incorporation inhibition test shown in Fig. 2; 10 μM for time dependent subsequent nucleotide hindrance test shown in Fig. S-2) for the next base incorporation. The reactions were quenched with 2.2 μL 100 mM EDTA at 7.5, 15, 30, and 60 min. Desalting of the reaction was performed with Oligo Clean & Concentrator kit (Zymo Research), resulting in ~ 10 μL purified aqueous RNA solutions. 1 μL of each solution was subjected to MALDI-TOF MS (Bruker ultrafleXtream) analysis. The remaining ~ 9 μL RNA products were used to test for exonuclease activity.

### Further elongation of C- or Azv-extended RNA.

The C-terminated RNA or Azvudine-terminated RNA (sequences shown in Fig. S-3a) was annealed by heating to 75°C for 3 min and cooling to room temperature in 1x RdRp buffer. Then, 5 μL of 4 μM SARS-CoV-2 Nsp12/Nsp7/Nsp8 (RdRp) complex was added to 5 μL of C-terminated RNA (C-RNA) or 5 μL of Azvudine terminated RNA (Azv-RNA) template-loop-primer solutions (4 μM) and incubate at room temperature for 5 min. Finally, 10 μL of 10 μM ATP and 10 μM GTP mixture in 1x RdRp buffer was added to the mixture of RdRp and RNA substrates and incubated at 30°C for 2 hr. The 20 μL extension reaction buffer contained 1 μM RNA substrate, 1 μM SARS-CoV-2 RdRp complex, 5 μM ATP and 5 μM GTP. Desalting of the reaction was performed with Oligo Clean & Concentrator kit (Zymo Research), resulting in ~ 10 μL purified aqueous RNA solutions. 1 μL of each solution was subjected to MALDI-TOF MS (Bruker ultrafleXtream) analysis. The remaining ~ 9 μL RNA products were used to test exonuclease activity.

### Comparison of SARS-CoV-2 exonuclease reaction for Azvudine (Azv)-terminated RNA with natural RNA in the same tube.

The C-extended RNA (2 μM) and Azv-extended RNA (2 μM) (sequences shown in Fig. 3a) were annealed by heating to 75°C for 3 min and cooling to room temperature in 1× exonuclease reaction buffer. After annealing, the two RNAs were mixed in equal volumes. To a 10 μL solution of 10 nM exonuclease complex (Nsp14/Nsp10) in 1× exonuclease reaction buffer and 10% DMSO, 10 μL annealed RNA mixture (1 μM each) was added and incubated at 37°C for 15 min. The final concentrations of reagents in the 20 μL reactions were 5 nM Nsp14/Nsp10 complex, 500 nM C-terminated RNA, 500 nM Azv-terminated RNA and 5% DMSO. After incubation for 15 min, each reaction was quenched by adding 2.2 μL of an aqueous solution of EDTA (100 mM). The 1x ExoN reaction buffer contains 50 mM pH = 8 Tris-HCl, 2 mM MgCl_2_ and 2 mM DTT. Following desalting using an Oligo Clean & Concentrator (Zymo Research), the samples were subjected to MALDI-TOF MS (Bruker ultrafleXtreme) analysis.

### Comparison of SARS-CoV-2 exonuclease reaction for full length Azvudine (Azv)-embedded RNA and natural RNA.

The fully extended Azv-embedded RNA (2 μM) and natural RNA (2 μM) produced from the RdRp extension experiment (sequences shown in Fig. 3c, e) were annealed by heating to 75°C for 3 min and cooling to room temperature in 1× exonuclease reaction buffer. To a 14 μL solution of 10 nM exonuclease complex (Nsp14/Nsp10) in 1× exonuclease reaction buffer and 1 uL DMSO, 5 μL of annealed RNA (2 μM each) was added and incubated at 37°C for 15 min. The final concentrations of reagents in the 20 μL reactions were 5 nM Nsp14/Nsp10 complex, 500 nM of fully extended Azv-embedded RNA or natural RNA, and 5% DMSO. After incubation for 15 min, each reaction was quenched by adding 2.2 μL of an aqueous solution of EDTA (100 mM). Following desalting using an Oligo Clean & Concentrator (Zymo Research), the samples were subjected to MALDI-TOF MS (Bruker ultrafleXtreme) analysis.

### Production of Azvudine-embedded RNAs with different subsequent nucleotides.

Different Azv terminated RNAs (5’-UUUUCUGCGCGUAGUUUUCAUCGCGAzv-3’, 5’- UUUUCAGCGCGUAGUUUUCAUCGCGAzv-3’, 5’-UUUUCCGCGCGUAGUUUUCAUCG-CGAzv-3’ or 5’-UUUUCGGCGCGUAGUUUUCAUCGCGAzv-3’) were annealed by heating to 75°C for 3 min and cooling to room temperature in 1x RdRp buffer. Then, 5 μL of 4 μM SARS-CoV-2 Nsp12/Nsp7/Nsp8 (RdRp complex) was added to 5 μL annealed RNA template-loop-primer solution (10 μM) and incubated at room temperature for 5 min. Finally, 10 μL of 20 μM corresponding NTP for subsequence nucleotide incorporation in 1x RdRp buffer was added to the mixture of RdRp and RNA template-loop-primer and incubated at 30°C for 2 hr. The 20 μL extension reaction buffer contained 1 μM RNA substrate, 1 μM SARS-CoV-2 RdRp complex, and 10 μM corresponding NTP. Desalting of the reaction was performed with an Oligo Clean & Concentrator kit (Zymo Research), resulting in ~ 10 μL purified aqueous RNA solutions. 1 μL of each solution was subjected to MALDI-TOF MS (Bruker ultrafleXtream) analysis. The remaining ~ 9 μL RNA products were used to test exonuclease activity.

### Comparison of SARS-CoV-2 exonuclease reaction for Azv + 1 RNAs and natural RNA.

The Azv + 1 RNA (2 μM) and same length natural RNA (2 μM) produced from RdRp extension experiments (sequences shown in Fig. 4 and S4) were annealed by heating to 75°C for 3 min and cooling to room temperature in 1× exonuclease reaction buffer. To a 14 μL solution of 10 nM exonuclease complex (Nsp14/Nsp10) in 1× exonuclease reaction buffer and 1 μL DMSO, 5 μL of annealed RNA (2 μM each) was added and incubated at 37°C for 15 min. The final concentrations of reagents in the 20 μL reactions were 5 nM Nsp14/Nsp10 complex, 500 nM of Azv + 1 RNA or natural RNA, and 5% DMSO. After incubation for 15 min, each reaction was quenched by adding 2.2 μL of an aqueous solution of EDTA (100 mM). Following desalting using an Oligo Clean & Concentrator (Zymo Research), the samples were subjected to MALDI-TOF MS (Bruker ultrafleXtreme) analysis.

## Supplementary Material

Supplementary Files

This is a list of supplementary files associated with this preprint. Click to download.

- AZVSupplementary492026.pdf

## Data Availability

All data are contained within the article and the Supplementary file.
